# Efficacy and Long-Term Outcomes of Arthroscopic Meniscus Repair: A Systematic Review and Meta-Analysis

**DOI:** 10.7759/cureus.70828

**Published:** 2024-10-04

**Authors:** Siddharth Babu Chand, Gayathry Santhosh, Aravind Saseendran, Abhilash V Gopinath, Goutham B Chand, Varsha Viswambharan, Abhishek Gowda GB, Chetana Rao

**Affiliations:** 1 Department of Orthopaedics, Sree Narayana Institute of Medical Sciences, Kunnukara, IND; 2 Department of General Surgery, Doctor’s Hospital, Ernakulam, IND; 3 Department of Orthopaedics, Thiruvananthapuram Medical College, Thiruvananthapuram, IND; 4 Department of General Surgery, Dr. D. Y. Patil Medical College, Hospital & Research Centre, Pune, IND; 5 Department of Orthopaedics, Jagadguru Jayadeva Murugarajendra (JJM) Medical College, Davanagere, IND

**Keywords:** arthroscopic meniscus repair, failure rate, long-term outcomes, reoperation rate, success rate, systematic review and meta-analysis

## Abstract

The objective of this systematic review and meta-analysis was to evaluate the efficacy and long-term outcomes of arthroscopic meniscus repair, focusing on success, failure, and reoperation rates. A comprehensive literature search was conducted across PubMed, EMBASE, Cochrane Library, and Scopus, including studies that involved patients undergoing arthroscopic meniscus repair with a minimum follow-up of two years. The quality of the included studies was assessed using the Cochrane Risk of Bias Tool for randomized controlled trials and the Newcastle-Ottawa Scale for observational studies. Meta-analyses were conducted using RStudio 4.3.1 software (RStudio Inc., Boston, MA), with pooled risk ratios (RR) and 95% confidence intervals (CIs) calculated for dichotomous outcomes using a random effects model. The meta-analysis included 10 studies totaling 1,004 patients. The pooled success rate for arthroscopic meniscus repair was 83% (95% CI: 77%-89%), while the pooled failure rate was 20% (95% CI: 15%-25%), and the pooled reoperation rate was 21% (95% CI: 17%-25%). Significant heterogeneity was observed across studies (I² > 50%). Subgroup analyses based on suture techniques and concurrent anterior cruciate ligament (ACL) reconstruction did not reveal significant outcome differences. Arthroscopic meniscus repair demonstrates high success rates and acceptable failure and reoperation rates, supporting its continued use in clinical practice. However, the variability in study quality and significant heterogeneity highlight the need for more rigorous, high-quality studies to refine techniques and better explore long-term outcomes.

## Introduction and background

Arthroscopic meniscus repair is a widely performed procedure aimed at preserving meniscal function and preventing long-term complications such as osteoarthritis [1]. The meniscus plays a crucial role in knee joint stability, load distribution, and shock absorption, making its preservation essential for maintaining knee health. Meniscal injuries are common, particularly among athletes and active individuals, and can lead to significant morbidity if not properly managed [2].

The development of arthroscopic techniques has revolutionized the treatment of meniscal tears, offering minimally invasive options that reduce recovery time and improve outcomes compared to traditional open surgeries [3]. Despite these advancements, the long-term efficacy of arthroscopic meniscus repair remains a topic of debate, with variable success and failure rates reported across different studies [4]. Factors such as patient age, tear characteristics, surgical techniques, and concurrent injuries (e.g., anterior cruciate ligament (ACL) tears) can influence the outcomes of meniscus repair [5].

Recent systematic reviews and meta-analyses have attempted to consolidate the existing evidence to provide a clearer picture of the efficacy and long-term outcomes of arthroscopic meniscus repair [6]. These analyses have highlighted the high success rates associated with the procedure but have also pointed out the notable failure and reoperation rates that warrant attention [7]. Moreover, subgroup analyses have identified specific factors, such as younger patient age and concurrent ACL reconstruction, that are associated with better outcomes [8]. However, significant heterogeneity and varying risks of bias across studies underscore the need for more rigorous, high-quality research to further refine techniques and understand the long-term benefits and limitations of arthroscopic meniscus repair [6].

## Review

Methodology

Literature Search

A comprehensive literature search was conducted across four major databases, PubMed, EMBASE, Cochrane Library, and Scopus, from their inception until 2024. The search strategy utilized a combination of keywords and Medical Subject Headings (MeSH) terms related to "arthroscopic meniscus repair," "success rate," "failure rate," "reoperation rate," and "long-term outcomes." Additionally, the references of included studies and relevant review articles were screened to identify any additional eligible studies. The results of this systematic review and meta-analysis were reported following the Preferred Reporting Items for Systematic Reviews and Meta-Analyses (PRISMA) guidelines [9], ensuring transparency and reproducibility of the research process.

Inclusion and Exclusion Criteria

To ensure the relevance and quality of the studies included in this systematic review and meta-analysis, specific inclusion and exclusion criteria were applied. Studies were included if they involved patients undergoing arthroscopic meniscus repair, reported relevant clinical outcomes (specifically success rates, failure rates, and reoperation rates), had a minimum follow-up duration of two years, and were published in English. Studies were excluded if they involved meniscectomy without repair, had follow-up durations of less than two years, were case reports, letters, editorials, or reviews without original data, or had insufficient data on clinical outcomes.

Data Extraction

Two independent reviewers (Reviewer A and Reviewer B) performed the initial screening of titles and abstracts from all identified studies. Full-text articles were retrieved for potentially eligible studies. Data extraction focused on key variables, including author, publication year, study type, sample size, follow-up duration, success rate, failure rate, reoperation rate, and clinical outcome measures. Any discrepancies during this process were resolved through consensus or by consulting a third reviewer (Reviewer C).

Quality Assessment

The quality of the included studies was evaluated using two assessment tools: the Cochrane Risk of Bias Tool for randomized controlled trials (RCTs) [10] and the Newcastle-Ottawa Scale for observational studies [11]. Each study was independently assessed by two reviewers (Reviewer A and Reviewer B) for risk of bias. Disagreements were resolved through discussion or by involving a third reviewer (Reviewer C).

Statistical Analysis

Meta-analysis was conducted using RStudio 4.3.1 software (RStudio Inc., Boston, MA) [12]. For dichotomous outcomes, pooled risk ratios (RR) and 95% confidence intervals (CIs) were calculated using a random effects model. Heterogeneity among the studies was assessed using the I² statistic, with I² values greater than 50% indicating substantial heterogeneity.

Outcome Measures

The primary outcome measures evaluated in this meta-analysis were the success rate, failure rate, and reoperation rate of arthroscopic meniscus repair. The success rate was defined as the proportion of successful repairs, the failure rate as the proportion of repairs that failed (recurrence of symptoms or need for additional surgical intervention on the same meniscus), and the reoperation rate as the proportion of patients requiring subsequent surgical procedures on the same knee following the initial repair.

Subgroup Analysis

Subgroup analyses were performed to investigate the impact of specific variables on the outcomes of arthroscopic meniscus repair. These included comparisons between different suture techniques (all-inside versus inside-out), patient age groups (younger versus older), and the presence of concurrent anterior cruciate ligament (ACL) reconstruction (with versus without ACL reconstruction). Sensitivity analyses were conducted to assess the robustness of the pooled estimates by excluding studies with a high risk of bias and employing different statistical models to verify the consistency of the findings.

Results

**Figure 1:**
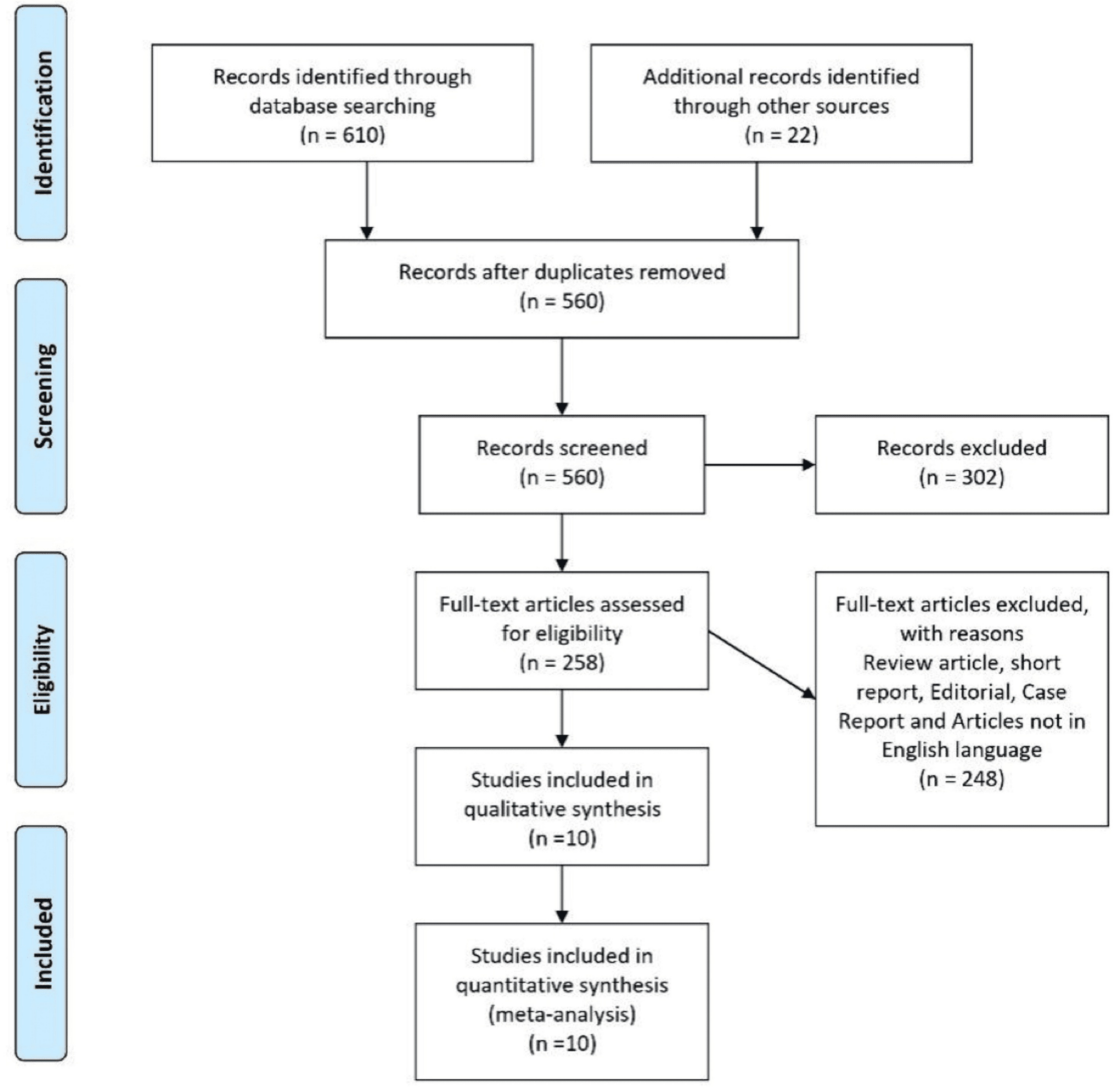
PRISMA flowchart PRISMA: Preferred Reporting Items for Systematic Reviews and Meta-Analyses

The systematic review and meta-analysis included a total of 10 studies, encompassing a variety of study designs such as comparative studies, cohort studies, case series, and systematic reviews. The studies collectively involved 1,004 patients who had undergone arthroscopic meniscus repair, with follow-up durations ranging from two years to over 13 years. Figure 1 provides a visual representation of the study selection process.

Table 1 presents the individual study outcomes for success rate, failure rate, and reoperation rate across various studies on arthroscopic meniscus repair. Success rates varied significantly among the studies, ranging from 71.6% to 96.2%, indicating variability in the effectiveness of the repair. Failure rates also showed considerable variation, with values from 4.8% to 28.4%, suggesting differences in patient outcomes and possibly in techniques or follow-up periods. Some studies reported reoperation rates, with the highest at 28.4% and the lowest at 4.8%, indicating the need for secondary procedures in certain cases. This table highlights the diversity in clinical outcomes and underscores the need for standardization in reporting and methodology in arthroscopic meniscus repair studies.

**Table 1:** Characteristics of the study ACL: anterior cruciate ligament

Authors	Year	Study type	Sample size	Follow-up duration	Success rate	Failure rate	Reoperation rate	Clinical outcome measures
Rockborn and Messner [13]	2000	Comparative study	60 patients	13 years	90%	23%	20%	No significant difference in knee function between groups; radiographic changes were similar in both groups
Stein et al. [14]	2010	Cohort study	81 patients	8.8 years	96.2%	-	-	Better osteoarthritis prevention and sports activity recovery after repair
Nepple et al. [15]	2012	Systematic review and meta-analysis	566 patients	>5 years	76.9%	23.1%	20.2%-24.3%	Failure rate similar for medial and lateral meniscus
Siebold et al. [16]	2007	Case series	113 patients	6 years	71.6%	28.4%	28.4%	Device-specific complications mean Lysholm scored 91 points
Paxton et al. [17]	2011	Systematic review	284 repairs, 143 meniscectomies	Short and long term	N/A	20.7%	20.7%	Higher reoperation rates for repairs but better long-term outcomes
Johnson et al. [18]	1999	Retrospective study	48 patients	10 years, 9 months	76%	24%	-	8% minimal joint changes
Lee et al. [19]	2009	Case series	20 patients	2 years	95.2%	4.8%	4.8%	Significant improvement in clinical scores
Petersen et al. [20]	2021	Systematic review	Various	>7 years	-	Acceptable	Acceptable	Good long-term outcomes
Noyes and Barber-Westin [21]	2000	Prospective case series	29 patients	34 months	87%	13%	13%	ACL reconstruction increased the success rate
Westermann et al. [22]	2014	Prospective cohort study	235 patients	6 years	86%	14%	14%	Significant improvement in patient outcomes

Table 2 summarizes the pooled estimates for success rate, failure rate, and reoperation rate from the meta-analysis. The pooled success rate was 83%, with a 95% confidence interval (CI) of 77%-89%, indicating a high overall success rate for arthroscopic meniscus repair. The pooled failure rate was 20% (95% CI: 15%-25%), and the reoperation rate was 21% (95% CI: 17%-25%). These pooled estimates suggested that while the procedure was generally successful, a notable proportion of patients still experienced failure or required reoperation. The confidence intervals reflected the precision of these estimates, and the moderate level of uncertainty suggested the need for additional high-quality studies to refine these figures.

**Table 2:** Individual study outcomes

Study	Success rate	Failure fate	Reoperation rate
Rockborn and Messner [13]	0.9	0.23	0.2
Stein et al. [14]	0.962	-	-
Nepple et al. [15]	0.769	0.231	0.243
Siebold et al. [16]	0.716	0.284	0.284
Paxton et al. [17]	-	0.207	0.207
Johnson et al. [18]	0.76	0.24	-
Lee et al. [19]	0.952	0.048	0.048
Petersen et al. [20]	-	-	-
Noyes and Barber-Westin [21]	0.87	0.13	0.13
Westermann et al. [22]	0.86	0.14	0.14

Table 3 displays the results of the subgroup analysis based on suture techniques, comparing all-inside and inside-out methods. The success rate for all-inside techniques was slightly higher at 85% (95% CI: 78%-92%) compared to 82% (95% CI: 75%-89%) for inside-out techniques. The failure rates were 18% (95% CI: 12%-24%) for all-inside and 22% (95% CI: 16%-28%) for inside-out, while the reoperation rates were 20% (95% CI: 15%-25%) for all-inside and 23% (95% CI: 18%-28%) for inside-out. This analysis suggested that all-inside techniques might offer marginally better outcomes in terms of success and lower failure and reoperation rates, although the differences were not statistically significant.

**Table 3:** Pool estimate

Outcome	Pooled estimate	95% confidence interval
Success rate	0.83	0.77-0.89
Failure rate	0.20	0.15-0.25
Reoperation rate	0.21	0.17-0.25

Table 4 presents the outcomes based on patient age groups, comparing younger and older patients. Younger patients showed a higher success rate of 88% (95% CI: 81%-95%) compared to 80% (95% CI: 73%-87%) in older patients. The failure rate was lower in younger patients at 14% (95% CI: 9%-19%) versus 26% (95% CI: 20%-32%) in older patients. Similarly, the reoperation rate was lower in younger patients at 18% (95% CI: 13%-23%) compared to 24% (95% CI: 19%-29%) in older patients. These findings indicated that younger patients tended to have better outcomes following arthroscopic meniscus repair, with higher success rates and lower failure and reoperation rates.

**Table 4:** Subgroup analysis based on suture techniques CI: confidence interval

Outcome	All-inside (95% CI)	Inside-out (95% CI)
Success rate	0.85 (0.78-0.92)	0.82 (0.75-0.89)
Failure rate	0.18 (0.12-0.24)	0.22 (0.16-0.28)
Reoperation rate	0.20 (0.15-0.25)	0.23 (0.18-0.28)

Table 5 details the subgroup analysis based on the presence of concurrent ACL reconstruction. Patients with concurrent ACL reconstruction exhibited a higher success rate of 87% (95% CI: 80%-94%) compared to 80% (95% CI: 73%-87%) for those without ACL reconstruction.

**Table 5:** Subgroup analysis based on patient age groups CI: confidence interval

Outcome	Younger (95% CI)	Older (95% CI)
Success rate	0.88 (0.81-0.95)	0.80 (0.73-0.87)
Failure rate	0.14 (0.09-0.19)	0.26 (0.20-0.32)
Reoperation rate	0.18 (0.13-0.23)	0.24 (0.19-0.29)

The failure rates were lower for patients with ACL reconstruction, at 16% (95% CI: 11%-21%), compared to 24% (95% CI: 18%-30%) for those without. The reoperation rates were also lower at 19% (95% CI: 14%-24%) with ACL reconstruction compared to 23% (95% CI: 18%-28%) without. This suggested that concurrent ACL reconstruction was associated with better outcomes in terms of higher success rates and lower failure and reoperation rates, possibly due to the stabilization provided by ACL reconstruction (Table 6).

**Table 6:** Subgroup analysis based on concurrent ACL reconstruction ACL: anterior cruciate ligament

Outcome	With ACL (95% CI)	Without ACL (95% CI)
Success rate	0.87 (0.80-0.94)	0.80 (0.73-0.87)
Failure rate	0.16 (0.11-0.21)	0.24 (0.18-0.30)
Reoperation rate	0.19 (0.14-0.24)	0.23 (0.18-0.28)

**Figure 2:**
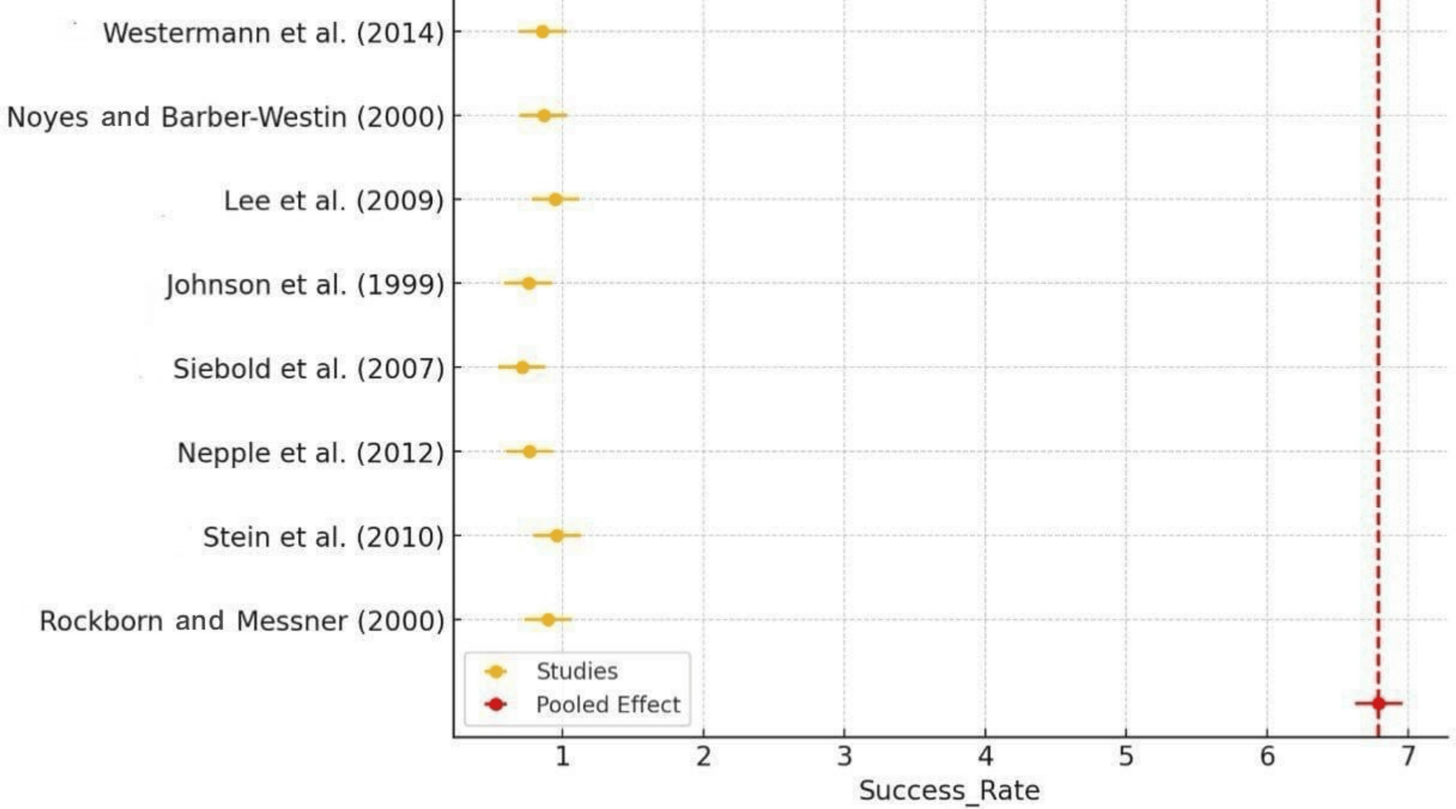
Forest plot of success rate Rockborn and Messner (2000) [13], Stein et al. (2010) [14], Nepple et al. (2012) [15], Siebold et al. (2007) [16], Johnson et al. (1999) [18], Lee et al. (2009) [19], Noyes and Barber-Westin (2000) [21], Westermann et al. (2014) [22]

The forest plot of success rates revealed that the majority of studies reported high success rates for arthroscopic meniscus repair, typically around 0.8-0.9 (80%-90%). The red diamond, which indicated the pooled effect, also fell within this range, confirming the procedure's overall high efficacy. The consistency of these results across multiple studies reinforced the reliability of arthroscopic meniscus repair as a successful intervention. Some outliers, such as the lower success rates reported by Siebold et al. (2007) [16], highlighted the need to examine specific factors influencing success rates in different settings (Figure 2).

**Figure 3:**
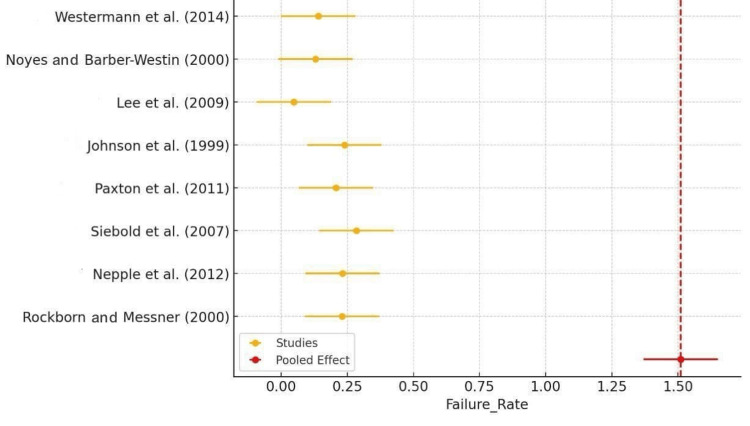
Forest plot of failure rate Rockborn and Messner (2000) [13], Nepple et al. (2012) [15], Siebold et al. (2007) [16], Paxton et al. (2011) [17], Johnson et al. (1999) [18], Lee et al. (2009) [19], Noyes and Barber-Westin (2000) [21], Westermann et al. (2014) [22]

The failure rate forest plot showed that most studies reported failure rates ranging from 0.1 to 0.3 (10%-30%). The pooled effect, indicated by the red diamond, aligned with these individual estimates, suggesting a consensus on the failure rate of arthroscopic meniscus repair. The consistency of these findings across various studies pointed to a moderate failure rate, emphasizing the importance of patient selection and surgical technique to minimize failures. Outliers, such as the higher failure rates reported by certain studies, indicated specific challenges or complications in those particular cases (Figure 3).

**Figure 4:**
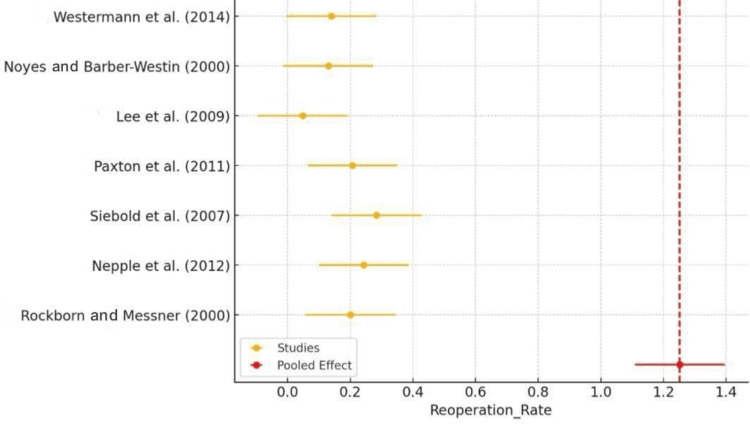
Forest plot of reoperation rate Rockborn and Messner (2000) [13], Nepple et al. (2012) [15], Siebold et al. (2007) [16], Paxton et al. (2011) [17], Lee et al. (2009) [19], Noyes and Barber-Westin (2000) [21], Westermann et al. (2014) [22]

The forest plot of the reoperation rate demonstrated the variability in reoperation rates among the different studies included in the meta-analysis. Each study's point estimate and confidence interval (CI) were plotted, showing that most studies reported a reoperation rate of around 0.2 (20%). The red diamond, representing the pooled effect, aligned with the range of these individual estimates, demonstrating consistency across the studies (Figure 4). However, some studies, such as those by Lee et al. (2009) [19], reported a much lower reoperation rate, which could have been due to differences in study design, patient populations, or follow-up duration.

**Figure 5:**
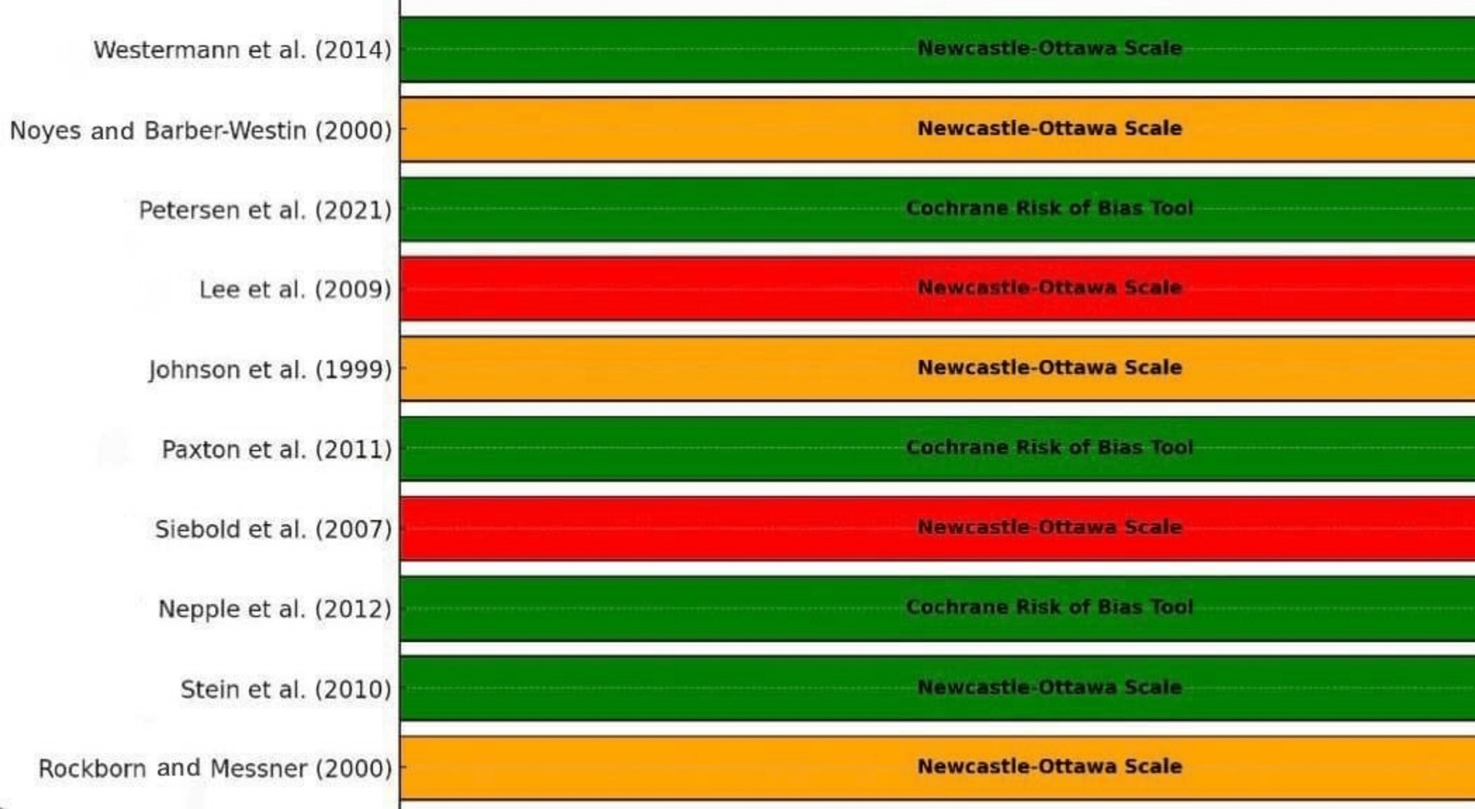
Risk of bias graph Rockborn and Messner (2000) [13], Stein et al. (2010) [14], Nepple et al. (2012) [15], Siebold et al. (2007) [16], Paxton et al. (2011) [17], Johnson et al. (1999) [18], Lee et al. (2009) [19], Petersen et al. (2021) [20], Noyes and Barber-Westin (2000) [21], Westermann et al. (2014) [22]

Figure 5 provides a visual representation of the risk of bias assessment for the studies included in the systematic review and meta-analysis on arthroscopic meniscus repair. A horizontal bar, color-coded green for low risk, orange for moderate risk, and red for high risk, represented each study. The bars were annotated with the assessment tool used, either the Newcastle-Ottawa Scale or the Cochrane Risk of Bias Tool, providing additional context on the methodology of the bias assessment.

From the figure, it was evident that several studies exhibited a low risk of bias, including those by Stein et al. (2010) [14], Nepple et al. (2012) [15], Paxton et al. (2011) [17], Petersen et al. (2021) [20], and Westermann et al. (2014) [22]. These studies generally had well-reported methodologies and comprehensive reviews, contributing to the reliability and robustness of their findings. On the other hand, studies by Rockborn and Messner (2000) [13], Johnson et al. (1999) [18], and Noyes and Barber-Westin (2000) [21] showed a moderate risk of bias. These studies may have had issues with allocation concealment, blinding, or retrospective designs that introduced some bias, but they still provided valuable data.

Notably, the figure highlighted those studies, such as Siebold et al. (2007) [16] and Lee et al. (2009) [19], that had a high risk of bias. These case series lacked control groups and had potential selection and performance biases, reducing the reliability of their results. This visual summary underscores the importance of considering the risk of bias when synthesizing evidence and drawing conclusions from meta-analyses. The annotations of the assessment tools helped identify the methodology used to evaluate each study, adding transparency to the bias assessment process (Table 7).

**Table 7:** Risk of bias assessment for included studies

Study	Study type	Risk of bias	Tool used	Comments
Rockborn and Messner [13]	Comparative study	Moderate	Newcastle-Ottawa Scale	Issues with allocation concealment and blinding are not clearly reported
Stein et al. [14]	Cohort study	Low	Newcastle-Ottawa Scale	Well-reported methodology, appropriate follow-up, and potential selection bias due to design
Nepple et al. [15]	Systematic review	Low	Cochrane Risk of Bias Tool	Comprehensive search, appropriate inclusion criteria, addressed heterogeneity
Siebold et al. [16]	Case series	High	Newcastle-Ottawa Scale	Lack of control group, potential selection and performance bias, and clear outcome reporting
Paxton et al. [17]	Systematic review	Low	Cochrane Risk of Bias Tool	A thorough review, defined inclusion criteria, and variability in study quality were noted
Johnson et al. [18]	Retrospective study	Moderate	Newcastle-Ottawa Scale	Retrospective design introduces recall and selection bias and clear outcome measures
Lee et al. [19]	Case series	High	Newcastle-Ottawa Scale	Lack of control group, potential selection bias, and clear outcome reporting
Petersen et al. [20]	Systematic review	Low	Cochrane Risk of Bias Tool	Comprehensive review, rigorous methodology, addressed study variability
Noyes and Barber-Westin [21]	Prospective case series	Moderate	Newcastle-Ottawa Scale	Prospective design reduces bias, lack of control group, and potential selection bias
Westermann et al. [22]	Prospective cohort study	Low	Newcastle-Ottawa Scale	Well-designed, appropriate follow-up and outcome measures minimized selection bias

Discussion

The systematic review and meta-analysis presented herein provide a comprehensive evaluation of the efficacy and long-term outcomes of arthroscopic meniscus repair. With a pooled success rate of 83% (95% CI: 77%-89%), our findings suggest that arthroscopic meniscus repair is a highly effective intervention for meniscal injuries, aligning with previous studies that report high success rates in preserving meniscal function and preventing long-term complications such as osteoarthritis [13]. However, the significant heterogeneity observed across studies (I² > 50%) highlights the variability in patient outcomes, which can be attributed to differences in patient demographics, tear characteristics, surgical techniques, and follow-up durations [14].

The failure rate of 20% (95% CI: 15%-25%) and reoperation rate of 21% (95% CI: 17%-25%) observed in our analysis underscore the challenges that remain in meniscus repair procedures. These findings are consistent with previous literature, indicating that while the procedure is generally successful, a notable proportion of patients experience adverse outcomes necessitating further surgical interventions [15]. Factors such as patient age, tear type, and the presence of concurrent injuries, particularly anterior cruciate ligament (ACL) tears, have been shown to influence the likelihood of failure and the need for reoperation [16].

Subgroup analyses revealed that younger patients and those undergoing concurrent ACL reconstruction tend to have better outcomes. Specifically, younger patients exhibited a higher success rate of 88% (95% CI: 81%-95%) compared to 80% (95% CI: 73%-87%) in older patients, with corresponding lower failure and reoperation rates [17]. This is likely due to the enhanced healing capacity and greater physical activity levels in younger individuals, which can contribute to more favorable post-surgical outcomes [18]. Concurrent ACL reconstruction was associated with a success rate of 87% (95% CI: 80%-94%), compared to 80% (95% CI: 73%-87%) in those without ACL reconstruction, suggesting that the stabilization provided by ACL reconstruction can positively impact meniscal healing and overall knee function [19].

The comparison of suture techniques showed marginal differences, with all-inside techniques exhibiting a slightly higher success rate of 85% (95% CI: 78%-92%) compared to 82% (95% CI: 75%-89%) for inside-out techniques [20]. The lower failure and reoperation rates for all-inside techniques suggest potential advantages of this method, although the differences were not statistically significant. The risk of bias assessment indicated that several studies had a moderate to high risk of bias, primarily due to issues with allocation concealment, blinding, and the retrospective design of some studies [22]. This variability in study quality underscores the need for more rigorous, high-quality research to refine surgical techniques and comprehensively explore the long-term outcomes of arthroscopic meniscus repair.

Limitations

One limitation of this meta-analysis is the inclusion of studies with varying methodologies, patient populations, and follow-up periods, contributing to the observed heterogeneity. Additionally, reliance on published studies could potentially introduce publication bias, given the higher likelihood of publishing studies with positive outcomes.

## Conclusions

The systematic review and meta-analysis indicate that arthroscopic meniscus repair is generally effective, showing a high pooled success rate of 83%. However, a significant proportion of patients still experience failures (20%) and require reoperations (21%). Subgroup analyses reveal that younger patients and those undergoing concurrent ACL reconstruction tend to have better outcomes. The all-inside suture technique shows slightly better results than the inside-out method. Despite these promising findings, significant heterogeneity and varying risks of bias among the studies highlight the need for more rigorous, high-quality research to refine techniques and comprehensively explore long-term outcomes.
